# Supplementary material for: Altered expression of miRNA profile in peripheral blood mononuclear cells following the third dose of inactivated COVID-19 vaccine
Source: PeerJ. 2025 Jan 22;13:e18856. doi: 10.7717/peerj.18856 (PMC11760199; doi:10.7717/peerj.18856)
Supplement: Supplemental Information 1 [file peerj-13-18856-s001.docx]

**Miame Checklist**

**Part 1 Experiment description**

- Human peripheral blood mononuclear cells (PBMCs)

- Experimental variables (Before and after third dose of inactivated COVID-19 vaccine)

- n-count for each group is 6

**Part 2 Array design**

- SurePrint Human miRNA Microarray

- Array type miRNA

- 2549 probes for miRNA

- Each probe was 60 mer long

**Part 3 Samples**

- **RNA extraction protocol**

Use TRIzol RNA isolation protocol (Thermo Fisher)

- **RNA clean-up and quantitation protocol**

Use RNasey Mini Kit (Qiagen 74104). The absorbance of a diluted RNA sample is measured at 260 and 280 nm using a UV spectroscopy.

- **Labelling protocol**

Use miRNA Complete Labeling and Hyb Kit (Agilent 5190-0456) for labeling.

1. Add 500 ng of total RNA to a 1.5-mL microcentrifuge tube.
2. Add 2 μL of CIP (Calf Intestinal Phosphatase) Master Mix and incubate the reaction at 37°C for 30 minutes.
3. Add 2.8 μL of DMSO to each sample and incubate at 100°C for 5 to10 minutes.
4. Immediately transfer to ice-water bath.
5. Add 4.5 μL Ligation Master Mix (1 μL 10×T4 RNA ligase buffer, 3.0 μL Cyanine 3-pCp, 0.5 μL T4 RNA ligase) and incubate at 16°C for 2 hours.
6. Completely dry the 16°C labeling reaction using a vacuum concentrator with heater at 45 to 55°C.

**Part 4 Hybridizations**

Kit and Instruments:

miRNA Complete Labeling and Hyb Kit (Agilent 5190-0456)

Hybridization Chamber, stainless (Agilent G2534A)

Hybridization Chamber gasket slides (Agilent G2534-60003)

Hybridization oven (Agilent G2545A)

Hybridization oven rotator for Agilent Microarray Hybridization Chambers (Agilent G2530-60029)

- **Hybridization protocol**

1. Resuspend the dried sample in 18 μL nuclease-free water.
2. Add 4.5 μL 10× Gene Expression Blocking Agent and 2×Hi-RPM Hybridization Buffer.
3. Incubate at 100°C for 5 minutes and immediately transfer to an ice bath for 5 minutes.
4. Load a clean gasket slide into the Agilent SureHyb chamber base with the label facing up and aligned with the rectangular section of the chamber base. Ensure that the gasket slide is flush with the chamber base and is not ajar.
5. Slowly dispense all of the volume of hybridization sample onto the gasket well in a “drag and dispense” manner.
6. Slowly place an array “active side” down onto the SureHyb gasket slide, so that the “Agilent”-labeled barcode is facing down and the numeric barcode is facing up. Verify that the sandwich-pair is properly aligned.
7. Place the SureHyb chamber cover onto the sandwiched slides and slide the clamp assembly onto both pieces.
8. Hand-tighten the clamp onto the chamber.
9. Vertically rotate the assembled chamber to wet the gasket and assess the mobility of the bubbles.
10. Place assembled slide chamber in rotisserie in a hybridization oven set to 55°C. Set hybridization rotator to rotate at 20 rpm.
11. Hybridize at 55°C for 20 hours.

**Part 5 Measurements**

- **Scanning protocol**

Instrument: Agilent Microarray Scanner (Agilent G2505C)

1. Assemble the slides into a slide holder.
2. Place assembled slide holders into scanner carousel.
3. Verify scan settings for color scans.

|  | Parameters |
| --- | --- |
| Dye channel | G (green) |
| Scan region | Agilent HD (61 x 21.6 mm) |
| Scan resolution | 3μm |
| Tiff file dynamic range | 20bit |
| Green PMT gain | 100% |

1. Click Scan Slot m-n on the Scan Control main window where the letter m represents the Start slot where the first slide is located and the letter n represents the End slot where the last slide is located.

- **Data extraction protocol to collect chip signal**

Software: Agilent Feature Extraction Software

Procedure:

1. Open the Agilent Feature Extraction (FE) software.
2. Add the images (.tif) to be extracted to the FE Project.
3. Set FE Project Properties.
4. Check the Extraction Set Configuration.
5. Save the FE Project (.fep) by selecting File > Save As and browse for desired location.
6. Select Project > Start Extracting and export data to txt.

**Part 6 Normalization controls.**

**-Hypothessis**

There is an altered miRNA expression profile in PBMCs for volunteers before and after a homologous booster (third) dose of the inactivated COVID-19 vaccine.

**-Normalization method**

Microarray data were processed with quantile normalization with Agilent GeneSpring GX v12.1 software.

**- Control**

The controls are volunteers before a homologous booster (third) dose of the inactivated COVID-19 vaccine.
