# Supplementary material for: Altered expression of miRNA profile in peripheral blood mononuclear cells following the third dose of inactivated COVID-19 vaccine
Source: PeerJ. 2025 Jan 22;13:e18856. doi: 10.7717/peerj.18856 (PMC11760199; doi:10.7717/peerj.18856)
Supplement: Supplemental Information 4 [file peerj-13-18856-s004.doc]

### STROBE Statement—Checklist of items that should be included in reports of ***cohort studies***

|  | Item No | Recommendation |
| --- | --- | --- |
| **Title and abstract** | 1 | (*a*) Samples of peripheral blood mononuclear cells (PBMCs) were harvested from healthcare volunteers before and 1-2 weeks after the third dose of CoronaVac. Line 25-27 |
|  |  | (*b*) miRNA microarray analysis in a discovery cohort of 6 volunteers identified 25 miRNAs with differential expression. Subsequently, the expression of 6 miRNAs related to immune responses was examined in PBMCs from a validation cohort of 31 participants via qRT-PCR. Differential expression of miRNAs after booster dose was validated for miR-25-5p, miR-34c-3p, and miR-206. miR-25-5p, miR-34c-3p, and miR-206 expression after booster dose was correlated with the receptor binding domain (RBD)-specific antibody response. Bioinformatic analysis revealed that miR-25-5p, miR-34c-3p, and miR-206 may target multiple pathways regulating immune responses and inflammation. Line 27-34 |
| Introduction | | |
| Background/rationale | 2 | The effects of COVID-19 vaccination on miRNA profiles in immune cells are poorly understood. Line 69-70 |
| Objectives | 3 | The impact of inactivated COVID-19 vaccines on miRNA expression has not been documented. In this study, blood samples were obtained from volunteers before and after a homologous booster (third) dose of the inactivated vaccine [CoronaVac](https://covid19.trackvaccines.org/vaccines/7/). Differential expression of miRNAs in peripheral blood mononuclear cells (PBMCs) was examined via microarray and qRT-PCR. Line 72-76 |
| Methods | | |
| Study design | 4 | There were 6 and 31 volunteers in the discovery and validation cohorts, respectively. (Line 81) |
| Setting | 5 | This prospective observational study was conducted during the government-launched COVID-19 vaccination campaign in 2021. Thirty-seven health care workers from Children’s Hospital of Zhejiang University School of Medicine were enrolled into the study. Each of the participant had received two doses of inactivated vaccine CoronaVac with an interval of 28 days between February 23, 2021 and May 28, 2021. Enrolled subjects provided written informed consent and received a third homogeneous dose of CoronaVac at least 6 months after the second dose between November 16, 2021 and January 18, 2022. Line 82-86 |
| Participants | 6 | (*a*) Only two blood draws before and after vaccination. No follow-up is needed. Line 92-93 |
|  |  | (*b*) **N/A** |
| Variables | 7 | miRNA expression profile and RBD antibody before and after third dose of inactivated COVID-19 vaccine. Line 115-116, Line 135, and Line 139 |
| Data sources/ measurement | 8* | Measurement of miRNA profile analysis by microarray. Line 105-118  Measurement of miRNA levels by qRT-PCR. Line 119-138  Measurement of receptor binding domain (RBD)-specific IgG by ELISA. Line 139-148 |
| Bias | 9 | N/A |
| Study size | 10 | The participants willing to be enrolled into the study. Line 81 |
| Quantitative variables | 11 | Variables include miRNA levels and RBD-specific IgG before and after third dose of inactivated vaccines. Line 115-116, Line 135, and Line 139 |
| Statistical methods | 12 | (*a*) Statistical analysis was performed using GraphPad Prism 8.0.2 software. Mann–Whitney test was used to compare miRNA expression before and after booster dose. Data were presented as median and interquartile range (IQR). Correlations were examined by the Spearman’s correlation coefficient. The p value < 0.05 was regarded as statistically significant. Line 149-153 |
|  |  | (*b*) N/A |
|  |  | (*c*) N/A |
|  |  | (*d*) N/A |
|  |  | (*e*) N/A |
| Results | | |
| Participants | 13* | (a) Total 37 subjects at both miRNA analysis and (RBD)-specific IgG analysis. Line 163-165 |
|  |  | (b) N/A |
|  |  | (c) N/A |
| Descriptive data | 14* | (a) The characteristics of volunteers for the present study are displayed in Table 1. The discovery cohort enrolled 6 healthcare workers. Concurrently, 31 healthcare workers were enrolled into the validation cohort. The interval between the second and third homogeneous doses of CoronaVac was at least 6 months. The booster dose had only mild local and systemic adverse events. Line 163-167 |
|  |  | (b) N/A |
|  |  | (c) N/A |
| Outcome data | 15* | N/A |
| Main results | 16 | (*a*) First, Levels of miR-25-5p, miR-34c-3p, and miR-206 are altered after the booster dose. Second, miR-25-5p, miR-34c-3p, and miR-206 expression after booster dose is correlated with the production of RBD-specific IgG. Third, miR-25-5p, miR-34c-3p, and miR-206 regulate immune responses via multiple pathways. Furthermore, miR-25-5p, miR-34c-3p, and miR-206 are associated with target genes for immune response. Line 168-213 |
|  |  | (*b*) N/A |
|  |  | (*c*) N/A |
| Other analyses | 17 | N/A |
| Discussion | | |
| Key results | 18 | The present study examined miRNA profile in PBMCs after a homologous booster dose with inactivated SARS-CoV-2 vaccine. Our results showed that there was differential expression of miR-25-5p, miR-34c-3p, and miR-206 after the booster dose. miR-25-5p, miR-34c-3p, and miR-206 were correlated with the production of anti-RBD IgG. These miRNAs may target multiple pathways involving immune response. Line 226-230 |
| Limitations | 19 | The present study has its limitations. First, the sample size was small with 6 volunteers from the discovery cohort and 31 from validation cohort. A large sample size is needed to validate the potential biomarker candidates. Second, the time point for examining miRNA expression was performed at 1-2 weeks after booster dose. The durability of altered miRNA expression profile warrants further investigation. Third, this study is limited to in silico prediction of target genes and future experiments are needed to validate these target genes. Line 299-304 |
| Interpretation | 20 | The third dose of inactivated COVID-19 vaccine alters the expression of miR-25-5p, miR-34c-3p, and miR-206 in PBMCs. These mRNAs may regulate immune response via multiple pathways. Line 306-308 |
| Generalisability | 21 | miR-25-5p, miR-34c-3p, and miR-206 may have the potential to be used as biomarkers for both COVID-19 infection and vaccination. Line 261-262 |
| Other information | | |
| Funding | 22 | This work was supported by the Natural Science Foundation of Zhejiang Province (Grant No. LGF22H150010), the National Natural Science Foundation of China (Grant No. **82070074, 82272191, and** 82370080), the Fundamental Research Funds for the Central Universities (Grant No. 226202200060), and the Key R&D Program of Zhejiang (Grant No. 2022C03163), the Health Commission of Zhejiang Province (Grant No. 2024KY491), and the Health Science Commission of Shaoxing (Grant No. 2023SKY 100 and 2023SKY 103). Line 318-323 |

*Give information separately for exposed and unexposed groups.

**Note:** An Explanation and Elaboration article discusses each checklist item and gives methodological background and published examples of transparent reporting. The STROBE checklist is best used in conjunction with this article (freely available on the Web sites of PLoS Medicine at http://www.plosmedicine.org/, Annals of Internal Medicine at http://www.annals.org/, and Epidemiology at http://www.epidem.com/). Information on the STROBE Initiative is available at http://www.strobe-statement.org.
